# Rationale and Design of the Cancer Immunotherapy Evidence Living (CIEL) Library: A Continuously Updated Clinical Trial Database of Cancer Immunotherapies

**DOI:** 10.1002/hsr2.72221

**Published:** 2026-03-30

**Authors:** Kim Boesen, Julian Hirt, Pascal Düblin, Heinz Läubli, Benjamin Kassenda, Lars G. Hemkens, Perrine Janiaud

**Affiliations:** ^1^ Research Center for Clinical Neuroimmunology and Neuroscience (RC2NB) University Hospital Basel and University of Basel Basel Switzerland; ^2^ Department of Health Eastern Switzerland University of Applied Sciences St. Gallen Switzerland; ^3^ Institute of Health and Nursing Science, Medical Faculty Martin Luther University Halle‐Wittenberg Halle (Saale) Germany; ^4^ Department of Clinical Research University of Bern Bern Switzerland; ^5^ Department of Clinical Research University Hospital Basel and University of Basel Basel Switzerland; ^6^ Department of Medical Oncology University Hospital Basel and University of Basel Basel Switzerland

**Keywords:** cancer, databases, immunotherapy, systematic reviews

## Abstract

**Background and Aims:**

Immunotherapies for cancers are tested in large numbers of clinical trials. It is difficult for clinicians and researchers to stay current with the evidence, and traditional systematic reviews and clinical guidelines are not suited to ensure a continued overview of all trials and their results. To address this problem, we designed the Cancer Immunotherapy Evidence Living (CIEL) Library.

**Methods:**

We included planned, ongoing, and completed interventional trials of immunotherapies for cancer, regardless of trial design (e.g., randomization, blinding, and type of comparator). We systematically searched PubMed (for published reports) and ClinicalTrials.gov (for registered clinical trials). PubMed‐retrieved records were screened using the AI‐assisted software ASReview and manually extracted and curated. We imported data from ClinicalTrials.gov using the Clinical Trials Transformation Initiative database, which then requires further curation. The CIEL‐Library was implemented as a web application. It also contained the “Match My Patient” feature, a patient‐centered clinical decision support system, aiming to filter planned, ongoing, or completed trials based on four patient characteristics (disease staging, previous treatments, performance status, and location). We piloted our database with one type of cancer immunotherapy, the tumor‐infiltrating lymphocytes (TIL) transfer. The CIEL‐Library was a prototype and no further developments are planned.

**Conclusions:**

The CIEL‐Library offers a blueprint for a dynamic evidence synthesis infrastructure by providing a collection of clinical trials with curated trial characteristics and results. This blueprint may be applied across fields, specialties, and topics. The main challenges to making a database of clinical trials are the time and resources needed to populate it with curated and updated data. The CIEL‐Library project highlights the potential and the main limitations of designing trial databases intended to be used in routine care.

## 1 Introduction

Treatment options for cancer patients have substantially increased, especially in the field of immunotherapies. Immunotherapies for cancer include small molecule agents (e.g., IDO (indoleamine 2,3‐dioxygenase) inhibitors), checkpoint inhibitors (e.g., PD‐1 and CTLA4 inhibitors), and Adoptive Cell Therapies (ACT; e.g., Tumor‐Infiltrating Lymphocytes (TIL), Chimeric Antigen Receptor (CAR) T cells and engineered T cells, and therapeutic cancer vaccines, (e.g., dendritic and deoxyribonucleic acid (DNA) or messenger ribonucleic acid (mRNA)‐based vaccines)) [1, 2, 3, 4, 5, 6, 7].

The number of planned, ongoing, and completed clinical trials investigating immunotherapy has increased drastically. In 2018, 1388 new trials were initiated across all types of immunotherapies for cancer, and in 2022 this number rose to 2095 new trials [8, 9]. The most prolific immunotherapeutic option is CAR T‐cells with, in December 2020, 778 trials registered on ClinicalTrials.gov [10]. The US Food and Drug Administration (FDA) approved six CAR‐T cell products for different lymphoma, multiple myeloma, and leukemia indications between 2017 and 2022 [11]. Three therapeutic cancer vaccines have also received FDA approval: the Bacillus Calmette‐Guérin vaccine for bladder cancer in 1990, a dendritic cell‐based vaccine (sipuleucel‐T) for prostate cancer in 2010 [12], and an oncolytic herpes simplex virus (talimogene laherparepvec) for melanoma in 2022 [13].

Immunotherapies for cancer are a rapidly evolving field with an escalating literature. Thus, it is time‐consuming and laborious for researchers to get a comprehensive overview and to evaluate key trial characteristics. Similarly, in clinical settings, complex oncology patient cases are often discussed at interdisciplinary tumor board meetings to decide on the most evidence‐based and patient‐tailored treatment. Such clinical decisions face the same challenges in identifying the most relevant evidence. Tools supporting research and clinical needs to identify and appraise the most current evidence efficiently, reliably, and transparently are needed.

Treatment decision‐making builds on clinical expertise, patient preferences, and available evidence [14]. Databases of published research, such as PubMed, and clinical trial registries, such as ClinicalTrials.gov, have improved access to information about published and planned clinical trials. However, the number of clinical trials testing healthcare interventions increases exponentially, and it is impossible for clinicians to stay on top of the evidence [15]. Clinical guidelines and systematic reviews also cannot reflect the rapid pace of evidence generation. Systematic reviews may be outdated even before publication [16] or not kept up‐to‐date [17], likely because it is time and resource‐demanding to manually search, screen, assess, and extract data from new records.

An increasing number of tools and software exist to automatize and streamline literature screening and data extraction in the systematic review process [18, 19], but these tools largely root in the traditional systematic review as a “static” publication. In contrast to such static reviews, one potential solution is to design a database of clinical trials, automating as many steps as possible.

### 1.1 The Cancer Immunotherapy Evidence Living (CIEL) Library

We aimed to develop a web application for a comprehensive library of all related immunotherapy clinical trials (planned, ongoing, and completed) and corresponding results to guide clinical decisions and clinical research.

The CIEL‐Library should serve a triple purpose as (i) an exhaustive library of clinical trials; (ii) a research resource to highlight gaps in the clinical research agenda and evidence base; and (iii) a clinical decision support system [20, 21, 22], with potential direct implementation in the clinic. It was planned to have two main features; the *CIEL‐Library* and *Match My Patient.*

The *CIEL‐Library* is the actual database containing all identified trials assessing immunotherapies of interest. The library is searchable, and the trials can be filtered. For each trial, we present general characteristics and key details following the Population, Intervention, Comparator, and Outcome (PICO) framework and a direct link to the original record. All data underlying the CIEL‐Library is available in a structured format, ready to be used for research purposes, for example, meta‐epidemiological assessments of immunotherapy clinical trials. This may enable identification of research gaps and highlight strengths and limitations of the research literature.

The *Match My Patient* search function was intended as a patient‐centered clinical decision support system to help identify ongoing (i.e., to find a trial to enroll yourself or your patient) or completed (i.e., to find results for making a therapeutic decision) clinical trials based on patient characteristics. A clinical decision support system can be defined as a platform/software where patients with specific characteristics, for example, age, sex, biomarkers, and disease severity, are compared with a “knowledge base” to guide decision making [20]. Such knowledge bases can be clinical guidelines, records of previously treated patients, or, in our case, an exhaustive collection of clinical trials. See use case example in Box 1.

BOX 1 **Clinical use case of CIEL's *Match my patient* feature.**

1 An oncologist wants to find clinical evidence on the potential use of tumor‐infiltrating lymphocytes for a patient with advanced melanoma (TNM stage IV), who has already received one line of systemic treatment, and is fully active (performance status 0). Using *Match My Patient*, the oncologist can identify trials which match the patient characteristics (See Figure 4 for an example). At the subsequent tumor board meeting, the oncologist presents the available evidence using the CIEL‐Library web application live.

Abbreviations: TNM = Extent of the tumor (T), extent of spread to the lymph nodes (N), and presence of metastasis (M).

## 2 Methods

**Figure 1:**
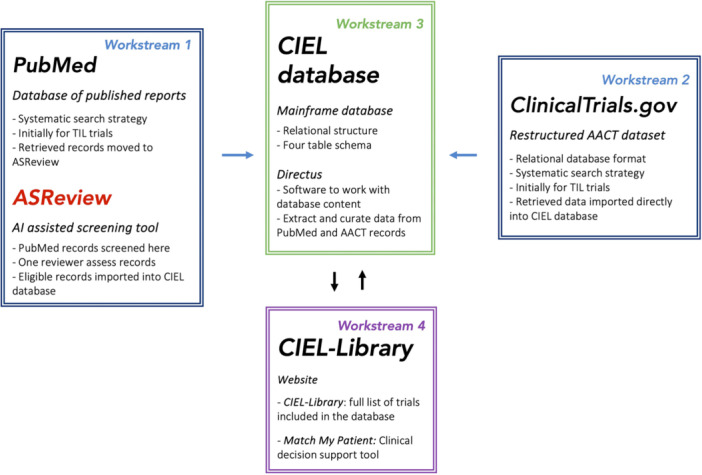
CIEL‐Library workstreams. AACT = aggregated analysis of ClinicalTrials.gov, AI = artificial intelligence, CIEL = cancer immunotherapy evidence living, TIL = tumor‐infiltrating lymphocytes.

In this rational and design paper, we highlight the CIEL‐Library's infrastructure and its four main workstreams: (i) Retrieval and screening of PubMed records, (ii) retrieval and screening of ClinicalTrials.gov records, (iii) designing the CIEL database, and (iv) designing the CIEL‐Library (Figure 1). We leveraged and optimized the implementation and methodology of previously established databases, also led by us on COVID‐19 trials [23], pragmatic trials [24, 25], and on FDA regulatory documents pertaining to cancer therapies approvals [26, 27]. The CIEL‐Library was a prototype, and no further development is planned. The project finished in 2024.

### 2.1 Eligibility Criteria

The CIEL‐Library aimed to contain any intervention study (trial) regardless of study phase, number of arms, randomization or not, blinding or not, and study design, assessing an immunotherapeutic intervention against any comparator (i.e., placebo, active drug, usual care, or no control) for any hematological or solid cancer. We designed and piloted the database with one specific type of immunotherapy, tumor‐infiltrating lymphocytes (TIL) transfer [28, 29]. TILs are immune cells extracted from patient tumor tissue and then expanded (grown) in‐vitro under specific circumstances for weeks before being re‐injected into the patients. TIL therapy has mainly been tested in metastatic melanoma patients [30] and non‐small cell lung cancer patients [31]. We chose this therapeutic option due to the anticipated number of trials (not too many, not too few) [32].

### 2.2 Data Sources and Search Strategy

Our strategy was to implement a systematic search approach for each data source, following best methodological practices from systematic reviews. We focused on PubMed as the source for published trial reports of results and ClinicalTrials.gov as the source for registered planned, ongoing, and completed trials. We assumed that these sources covered the most impactful research studies in the field.

Three authors (J.H., P.J., and K.B.) designed and verified a systematic search string with a pre‐emptive list of keywords for the condition (“any cancer disease”) and intervention (“tumor‐infiltrating lymphocytes”), see Supporting Information S1. Search terms were collected by expert consultation (B.K.) and initial searches on the topic using PubMed, Epistemonikos, International Prospective Register of Systematic Reviews (PROSPERO), Scopus, and Google Scholar. In addition, we identified controlled vocabulary using the Medical Subject Heading (MeSH)‐browser. PubMed PubReMiner [33] was also used to identify additional free text terms and controlled vocabulary used in relevant publications.

### 2.3 Identification and Screening of PubMed Records (Workstream 1)

To reduce the time needed to screen and identify relevant trials, we implemented a machine‐learning‐assisted screening tool, ASReview [34, 35]. It is an open‐source tool developed at Utrecht University, the Netherlands. It uses a machine‐learning algorithm to rank the references obtained from database searches according to their relevance. Instead of screening all references (in random order), ASReview presents the most relevant reference first, and the least relevant reference last.

For our initial search for tumor‐infiltrating lymphocyte trials, we searched PubMed and retrieved 14,004 records (May 2, 2023). We employed a two‐step screening process. We first restricted our total sample adding the PubMed filter “Clinical study” to our search and screened all 604 resulting records. We subsequently used the trained ASReview's algorithm on those 604 records to screen the full PubMed sample. We had predefined an arbitrary stopping rule of 100 consecutive non‐eligible records. Ultimately, we screened a total of 1476 records (11% of the total sample), and we identified 136 relevant records (0.97% of the total sample). We describe our use of ASReview in more detail elsewhere [36].

### 2.4 Identification and Screening of ClinicalTrials.gov Records (Workstream 2)

ClinicalTrials.gov is the largest trial registry with more than 426,000 interventional trials registered by November 2025. For data import, we used the Clinical Trials Transformation Initiative's database (Aggregated Analysis of ClinicalTrials.gov; AACT), which is a daily updated copy of all information on ClinicalTrials.gov made available in a relational database format [37, 38]. Compared to using ClinicalTrials.gov's native Application Programming Interface (API), the AACT's relational database format (in theory) allows a more comprehensive and smooth integration to import data directly into our CIEL database, in addition to also containing more structured data.

Practically, we downloaded the AACT data set as a static database dump on a monthly basis, consisting of 47 data tables in text file (.txt) format. The data dump is updated daily on AACT's website, and AACT also makes available a cloud‐based version of the database, which is also updated daily. We searched the tables “interventions.txt” and “interventions_other_names. txt.” using the same keywords derived from our PubMed literature search string (Supporting Information S1). The most recent search of the AACT text files (January 31, 2024) returned 270 trials. Retrieved records were automatically imported into the CIEL database and then checked for eligibility during data curation by one reviewer (P.J.), and uncertainties were discussed with a second reviewer (J.H.). Overall, 179 records (66%) were eligible and 91 records (34%) were not eligible, for example, not an interventional trial or not the right population, for example, treatment for psoriasis.

### 2.5 CIEL Database Architecture (Workstream 3)

We designed the underlying CIEL Database as a relational database. A relational database combines multiple tables (or spreadsheets) and allows cross‐referencing across these tables through unique identifiers [39]. In contrast to single‐sheet non‐relational tables (like Excel spreadsheets), this structure also allows to increase the database content.

**Figure 2:**
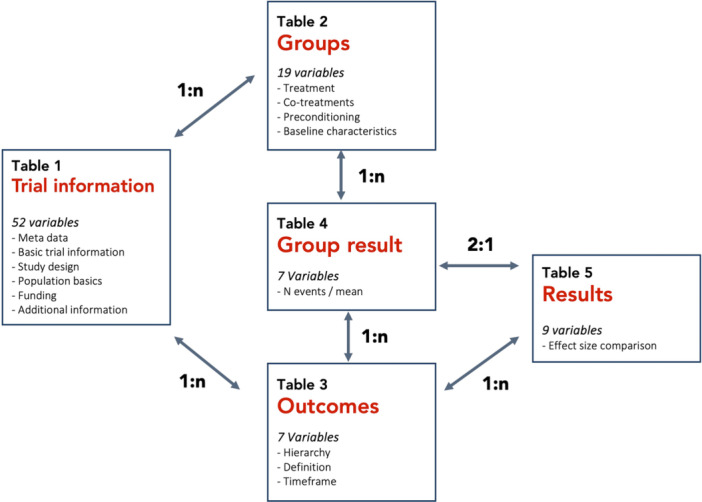
Overview of the CIEL‐library relational database.

Our software engineer (P.D.) built a relational Structured Query Language database using PostgreSQL as the relational database management system, which is an open‐source non‐proprietary program. We made a five‐table database schema illustrated in Figure 2 and the codebook is available as Supporting Information S2:

1. “Trial information”; 52 variables; 29 automatically imported from AACT; contains all basic trial information;
2. “Groups”; 19 variables; 4 automatically imported; contains all information related to study groups, for example, allocated treatments and also baseline information (provided it has been reported on a group level);
3. “Outcomes”; 7 variables; 4 automatically imported; describes the assessed and reported trial outcomes;
4. “Group results”; 7 variables; 5 automatically imported; contains results at the study group level; and
5. “Results”; 9 variables; 6 automatically imported; contains the corresponding comparison results. The codebook underlying the database is available from the Open Science Framework (OSF) [40], in which all 82 variables are specified, also those variables that did not appear on the web application.

We used the software application Directus [41] to access and curate the underlying database. Directus employs a user‐friendly graphical interface, and the application is browser‐based, making it easy to use, especially in collaborative setups. The Directus interface allows to extract and curate data from all included trial records retrieved from both PubMed and AACT.

### 2.6 Data Entry and Curation

The CIEL‐Library contained both automatically and manually extracted data. Metadata from PubMed retrieved publications, such as title, abstract, and year of publication, were automatically imported. Other trial information (e.g., trial design, outcomes, and results) originating from PubMed‐retrieved publications required manual extraction by full‐text assessment of the corresponding paper. Depending on the individual trial reporting, this may have yielded different levels of information completeness in the CIEL‐Library.

For ClinicalTrials.gov entry data, we were able to automatically import 46 variables from the AACT database. The main hindrance for automatic extraction of all variables was the available AACT data format. Some information on ClinicalTrials.gov is not coded in a format that allows automated identification and extraction through the AACT database. For example, when multiple interventions are administered in a trial they may be reported in the same data field without defining which intervention is the main treatment, which is co‐treatment, and so forth. Such information must therefore be curated manually after the automatic data import. Similarly, some trial information in AACT (e.g., contact person or group description), are moved from one table to another depending on whether trial results are reported or not, which adds to the complexity, and arguably confusion, of importing data effectively. The prototype did not cluster multiple records on the same trial or flag inconsistencies between multiple resources. Such features would require automated matching, even on outcome and results‐levels (which were not implemented in the prototype). Secondly, established hierarchies of the data sources should instruct the system about which data points to use [42].

For the initial database implementation, we prioritized results extraction for the “objective response rate” outcome [43]. This is a commonly used efficacy outcome reported in interventional trials based on the Response Criteria in Solid Tumor (RECIST) framework [44]. We did this for feasibility reasons to not overload the first manual data extraction phase; many different outcomes may be reported in cancer trial publications, and at multiple time points, which is time consuming to extract manually. Other efficacy outcomes and safety outcomes that are reported or specified on trial registries or publications were recorded in our database (see Figure 2 in Box “Table 3”; outcomes), but the results were not systematically extracted and curated (see Figure 2 in Boxes “Table 4” and “Table 5”; group result and results).

### 2.7 The CIEL‐Library Website and Web App (Workstream 4)

The CIEL‐Library website, accessible at http://ciel-library.org, provides an overview of the project. We created a web application containing the actual *CIEL‐Library* (i.e., the library of clinical trials) and *Match My Patient*. The code underlying the app is available on GitHub (https://github.com/rc2nb/ciel-library/tree/main/frontend). The web application landing page contained a high‐level dashboard showing information on the number of trials, patients, and interventions.

**Figure 3:**
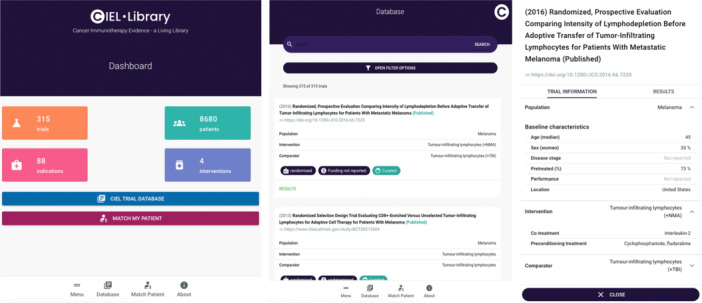
Overview of the CIEL‐Library. From left to right: landing page of the web App with a dashboard; view of the trials in the CIEL‐Library with search bar and filters option that can be accessed by clicking on “CIEL TRIAL DATABASE” in the previous panel; and a more granular view of the trial population that can be accessed by clicking on any trial of interest.

The *CIEL‐Library* enabled the user to search and filter all included immunotherapy trials based on six options (Figure 3): type of therapy (e.g., tumor‐infiltration lymphocytes), trial status (e.g., ongoing or completed), trial phase (e.g., phase 1 or 2), study design (e.g., randomized or non‐randomized), disease category (e.g., dermatology), and level of data curation (e.g., curated or automatically imported). The search results were presented as a list of relevant trials, with basic information for each trial visible including date of publication or registration, title, trial status (e.g., published or ongoing), link to full publication or registration entry, whether the trial is randomized or not, level of data curation, and funding. Furthermore, information regarding the *Population* (i.e., the patient group), *Intervention* (i.e., the tested treatment), and *Comparator* (the reference treatment, if any) was also shown. By clicking on the trial, more granular baseline characteristics (i.e., age, sex, disease stage, pre‐treatment, performance status, and location of the trial), intervention/comparator (i.e., concomitant and preconditioning treatments) and results information appeared. We used color coding to indicate whether results were reported (green—reported; grey—not reported).

Only some of the total variables collected were presented as to not overwhelm the user. The underlying database is available upon request, allowing a more granular assessment of each trial for research purposes.

**Figure 4:**
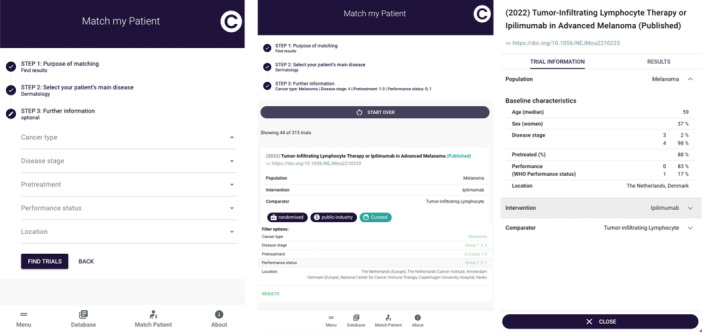
Overview of the Match My Patient feature. From left to right: Match My Patient search tool accessible from the dashboard; relevant trials identified with under each trial title an overview of the corresponding matching terms with the search highlighted in green; detail view of a relevant trial.

In *Match My Patient*, trials could be identified through a three‐step process (Figure 4). Step 1: Search for either *ongoing* trials (i.e., to find a trial to enroll your patient) or *completed* trials (i.e., to find results for making a therapeutic decision). Step 2: Select the patient's main disease category, for example, metastatic melanoma. Step 3: Specify patient characteristics; disease stage (stage 1–4; based on the extent of the tumor (T), extent of spread to the lymph nodes (N), and presence of metastasis (M) (TNM) staging system [45]), pre‐treatment status (treatment naïve; 1–3 previous cancer treatments, or 4+ cancer treatments), performance status (0–4, based on the Eastern Cooperative Oncology Group scale) [46], and geographic location of the patient. The resulting relevant trials were then presented as described above. Any search criteria matching a relevant trial were color‐coded in green (Figure 4).

## 3 Discussion

CIEL's semi‐automated extraction process embedded in a relational database framework sets it apart from static evidence synthesis products, like regular systematic reviews. Such reviews are rarely up‐to‐date and/or have narrowly defined scopes, for example, one intervention for one type of disease.

Compared to known clinical trial platforms [47, 48, 49], CIEL integrated some semi‐automated workstreams. The manual work required to extract and curate data from publications to ensure that the database is updated is a main limitation. It is resource‐demanding, and it would be challenging to sustain.

CIEL's potential clinical application and its use of clinical trials as the “knowledge base” have not, according to our knowledge, been described by other clinical decision support systems [21, 22]. Such systems have gained interest in recent years, especially in complex specialties such as oncology [50, 51]. CIEL's *Match My Patient* mimics this concept, except that CIEL's knowledge base consists of interventional clinical trials, which may (in theory) be a more granular knowledge base and enable more personalized treatment decisions. The methodological challenge of using CIEL's aggregated group‐level results to match individual patient characteristics should be acknowledged. However, this is the case for any individual treatment decision based on aggregate results.

### 3.1 CIEL's Next Phases

We want to clarify that CIEL will not be updated further due to a lack of funding. We have presented CIEL's blueprint, which may serve as inspiration for future projects. However, we would like to highlight what should (ideally) constitute the next phases. In addition to continued reiterative improvements of the above outlined workstreams, a technical and clinical validation phase is needed. A pilot testing of the CIEL‐Library web application should collect user information to validate (or refute) its usefulness as a clinical tool for supporting decision making. Secondly, additional important data sources could be added, including the World Health Organization International Clinical Trial Registry Platform and the Embase database of published literature. Less critical, albeit potentially useful, features would be the implementation of a ranking algorithm for the trials identified using the *Match My Patient* tool. A dataset download feature to allow dataset reuse for further research should also be added. Beyond this, automatizing manual workflows, such as a full‐scale integration of the PubMed API into ASReview to allow genuinely automated updates, would improve the database's long‐term sustainability. Large language models, such as ChatGPT, may open the possibilities of extracting otherwise unstructured text and data from journal publications into structured “machine‐readable” formats [52, 53]. Nevertheless, manual validation to ensure accuracy would be needed.

### 3.2 Limitations

Although the CIEL‐Library was not a fully automated living library, many parts of the machinery were implemented to allow for a continuously updated database. The main challenge to CIEL's viability and usefulness is the time and resources needed to manually extract, import, and curate data from heterogenous and incomplete data sources, both journal publications and trial registries. As stated already in 2001 [20], the prerequisite to designing a useful trial database is access to “machine‐readable” research literature. Twenty‐five years later, the AACT initiative is the best and only attempt at providing a structured clinical trial dataset, albeit limited to the data available on ClinicalTrials.gov. An additional challenge arises from data sources such as ClinicalTrials.gov that are themselves continuously updated. Changes in the registry over time can create discrepancies between previously extracted records and newer versions, necessitating version control, audit trails, and periodic human oversight to reconcile conflicts and maintain data integrity within the library.

Secondly, the CIEL‐Library output can only be as good and reliable as the included data, which pertains both to reporting issues and to the methodological quality of the included trials [20]. Reporting of phase 1 cancer trials frequently deviates between trial registry and final publication [54]; reporting of interventional phase 2 cancer trials in medical journals is, in general poor [55]; and harms reporting from phase 3 trials have been highlighted as being particularly poorly reported in journals [56] and deviating from trial registries and clinical study reports [57].

Journal publications are not reliable, exhaustive reports of trial information, and ideally, all information imported into CIEL should come from clinical trial registries. Due to the current publication paradigm, the lion's share of trial data, especially pertaining to phase I and II trials, is mostly available in journal publications. Clinical trial registries, and the AACT database, enables a large‐scale automated data import. However, trial registry entries are not perfect records of trial information, and results are often not reported [58, 59]. These issues pertain not only to cancer trials but are general issues of evidence‐based healthcare.

Thirdly, the methodological quality of the included immunotherapy trials may have limited CIEL's usefulness. It is well‐documented that pivotal cancer trials used for drug approvals have methodological limitations. These include the use of single‐arm designs without a comparator; the use of inappropriate and inferior comparators, reporting of surrogate outcomes and not patient‐reported quality of life and overall survival, and a high risk of bias [60, 61]. The CIEL‐Library could provide the data to investigate these issues in more detail, but CIEL did not include any literature appraisal features itself, such as risk of bias assessments.

## 4 Conclusions

The CIEL‐Library is a prototype that offers a blueprint for future evidence synthesis. The current main limitations are the technical challenges related to aggregating heterogenous data sources, the time required to extract information from paper‐based publications, and the general low reporting quality of the included trials.

The CIEL‐Library infrastructure is applicable across fields, indications, and specialties. We hope that the medical community will gradually become less focused on paper‐based publications and instead shift towards a more rigorous and complete trial registry reporting. Such an ideal reporting ecosystem would (theoretically) enable a fully automatable and machine‐readable clinical trial dataset, which could be directly integrated into databases such as the CIEL‐Library for the benefit of clinicians, policy makers, and patients.

## Author Contributions

**Kim Boesen:** conceptualization, data curation, investigation, methodology, project administration, validation, visualization, writing – original draft, writing – review and editing. **Julian Hirt:** conceptualization, methodology, validation, writing – original draft, writing – review and editing. **Pasca Düblin:** conceptualization, software, validation, writing – review and editing. **Heinz Läubli:** methodology, validation, writing – review and editing. **Benjamin Kassenda:** conceptualization, methodology, validation, writing – review and editing. **Lars G. Hemkens:** conceptualization, funding acquisition, methodology, project administration, resources, supervision, validation, writing – review and editing. **Perrine Janiaud:** conceptualization, funding acquisition, methodology, project administration, resources, supervision, validation, writing – original draft, writing – review and editing.

## Ethics Statement

The CIEL database, which we referred to in manuscript, contains—without exception—published and aggregated data. We did not actively collect data or store individual participant data. There was no expectation of vulnerability of any persons involved (no patients are involved). With regard to the Swiss Human Research Act, our research did not concern human diseases and the structure and function of the human body.

## Conflicts of Interest

RC2NB (Research Center for Clinical Neuroimmunology and Neuroscience Basel) is supported by the Foundation Clinical Neuroimmunology and Neuroscience Basel. RC2NB has a contract with Roche for a steering committee participation of L.G.H., unrelated to this work. K.B., J.H., P.D., B.K., L.G.H., and P.J. declare no conflicts of interest. H.L. received travel grants and consultant fees from BMS and Merck, Sharp and Dohme (MSD). H.L. received research support from BMS, GlycoEra, and Palleon Pharmaceuticals. H.L. is a co‐founder of Glycocalyx Therapeutics.

## Transparency Statement

The corresponding author, Perrine Janiaud, affirms that this manuscript is an honest, accurate, and transparent account of the study being reported; that no important aspects of the study have been omitted; and that any discrepancies from the study as planned (and, if relevant, registered) have been explained.

## Supporting information

Additional File 1.

Additional_File_2Codebook_CIEL.

## Data Availability

All information pertaining to the CIEL‐Library can be found on the CIEL website (https://ciel-library.org). All data underlying the CIEL‐Library are available upon request to the corresponding author. The code underlying the CIEL‐Library App is available on GitHub (https://github.com/rc2nb/ciel-library/tree/main/frontend).
