# Supplementary material for: Rationale and Design of the Cancer Immunotherapy Evidence Living (CIEL) Library: A Continuously Updated Clinical Trial Database of Cancer Immunotherapies
Source: Health Sci Rep. 2026 Mar 30;9(4):e72221. doi: 10.1002/hsr2.72221 (PMC13087519; doi:10.1002/hsr2.72221)
Supplement: Supplementary file 1 — Additional File 1. [file HSR2-9-e72221-s001.docx]

**Additional File 1**

1. PubMed search strategy

| # | Entry |
| --- | --- |
| 1 | (Cancer*[tiab] OR Carcinoma*[tiab] OR malign*[tiab] OR Malignan*[tiab] OR Neoplas*[tiab] OR neoplasms[MeSH] OR Oncolog*[tiab] OR Tumor*[tiab] OR Tumour*[tiab]) |
| 2 | ("antigen-specific T lymphocyte"[tiab] OR "antigen-specific T lymphocyte"[tiab] OR "antigen-specific T lymphocytes"[tiab] OR "antigen-specific T lymphocytes"[tiab] OR "antigen-specific T-lymphocyte"[tiab] OR "antigen-specific T-lymphocyte"[tiab] OR "antigen-specific T-lymphocytes"[tiab] OR "antigen-specific T-lymphocytes"[tiab] OR "Antitumor lymphocytes"[tiab] OR "Antitumour lymphocytes"[tiab] OR "Autologous TIL"[tiab] OR "Lymphocytes, tumor-infiltrating"[MeSH] OR "TIL therapy"[tiab] OR "TIL transfer"[tiab] OR "Tumor Derived Activated Cell"[tiab] OR "Tumor Derived Activated Cells"[tiab] OR "Tumor infiltrating lymphocyte"[tiab] OR "Tumor infiltrating lymphocytes"[tiab] OR "Tumor Infiltration Lymphocytes"[tiab] OR "Tumor-Derived Activated Cell"[tiab] OR "Tumor-Derived Activated Cells"[tiab] OR "Tumor-infiltrating lymphocyte"[tiab] OR "Tumor-infiltrating lymphocytes"[tiab] OR "Tumor-Infiltration Lymphocytes"[tiab] OR "Tumour infiltrating lymphocyte"[tiab] OR "Tumour infiltrating lymphocytes"[tiab] OR "Tumour Infiltration Lymphocytes"[tiab] OR "Tumour-infiltrating lymphocyte"[tiab] OR "Tumour-infiltrating lymphocytes"[tiab] OR "Tumour-Infiltration Lymphocytes"[tiab] OR "Young-TIL"[tiab] OR Lifileucel[tiab] OR TIL[tiab] OR TILs[tiab]) |
| 3 | 1# AND #2 |
| 4 | ("animals"[mesh] NOT "humans"[mesh]) |
| 5 | 3 NOT 4 |

2. ClinicalTrials.gov search strategy; searched via Clinical Trials Transformation Initiative’s database (AACT) using the data tables ‘interventions.txt’ and ‘intervention_other_names.txt’

"antigen-specific T lymphocyte" OR "antigen-specific T lymphocytes" OR "antigen-specific T-lymphocyte" OR "antigen-specific T-lymphocytes" OR "Antitumor lymphocytes" OR "Antitumour lymphocytes" OR "Autologous TIL" OR "TIL therapy" OR "TIL transfer" OR "Tumor Derived Activated Cell" OR "Tumor Derived Activated Cells" OR "Tumor infiltrating lymphocyte" OR "Tumor infiltrating lymphocytes" OR "Tumor Infiltration Lymphocytes" OR "Tumor-Derived Activated Cell" OR "Tumor-Derived Activated Cells" OR "Tumor-infiltrating lymphocyte" OR "Tumor-infiltrating lymphocytes" OR "Tumor-Infiltration Lymphocytes" OR "Tumour infiltrating lymphocyte" OR "Tumour infiltrating lymphocytes" OR "Tumour Infiltration Lymphocytes" OR "Tumour-infiltrating lymphocyte" OR "Tumour-infiltrating lymphocytes" OR "Tumour-Infiltration Lymphocytes" OR "Young-TIL" OR Lifileucel OR TIL OR TILs
